# Vascular Endothelial Growth Factor plus Epidermal Growth Factor Receptor Dual Targeted Therapy in Metastatic Colorectal Cancer: Synergy or Antagonism?

**DOI:** 10.1155/2009/937305

**Published:** 2009-12-06

**Authors:** John L. Marshall

**Affiliations:** Lombardi Comprehensive Cancer Center, Georgetown University, Washington, DC 20057, USA

## Abstract

There has been an intensive effort to develop novel therapies for the treatment of metastatic colorectal cancer (mCRC). The anti-epidermal growth factor receptor (EGFR) antibodies panitumumab and cetuximab and the anti-vascular endothelial growth factor (VEGF) antibody bevacizumab have demonstrated clinical efficacy and acceptable toxicity in the treatment of mCRC as single agents or in combination with chemotherapy. Recent clinical trials have explored the efficacy and safety of treatment regimens incorporating chemotherapy in combination with bevacizumab and either panitumumab or cetuximab in patients with mCRC. Results from the BOND-2 trial, which investigated cetuximab, bevacizumab, and chemotherapy in mCRC, provided support for this therapeutic approach. Two large randomized phase 3 trials were initiated to evaluate firstline treatment of mCRC. The Panitumumab Advanced Colorectal Cancer Evaluation (PACCE) study investigated the efficacy and safety of oxaliplatin- or irinotecan-based chemotherapy and bevacizumab with or without panitumumab; CAIRO2 assessed the efficacy and safety of capecitabine/oxaliplatin and bevacizumab with or without cetuximab. In both trials, the combination of bevacizumab, an EGFR-specific antibody, and chemotherapy in first-line treatment of mCRC was associated with increased toxicity and no improvement in patient outcome. These results suggest that these specific combinations should not be used in first-line mCRC outside investigational studies.

## 1. Introduction

Colorectal cancer is among the most common cancers in the United States, and it has been estimated that more than 50 000 patients died from colorectal cancer in 2007 [1]. Consequently, there is great interest in the development of novel therapies for the disease. In particular, recent studies have investigated the utility of treatment with targeted therapies in combination with chemotherapy in metastatic colorectal cancer (mCRC), with the aim of improving antitumor activity while maintaining acceptable toxicity. Moreover, there has been a belief that therapeutic approaches using a combination of targeted therapies plus chemotherapy might result in even greater efficacy [2]. In clinical studies, treatment with panitumumab, a fully human immunoglobulin (Ig) G2 monoclonal antibody targeting the epidermal growth factor receptor (EGFR), or cetuximab, a chimeric IgG1 monoclonal antibody targeting the EGFR, in combination with chemotherapy has been shown to have antitumor activity and be well tolerated in mCRC [3–7]. Additionally, panitumumab [8–10] and cetuximab [5, 11] have proven to be effective as single agents for the treatment of mCRC in patients refractory to first-line treatment. Furthermore, treatment with bevacizumab, a monoclonal antibody against vascular endothelial growth factor (VEGF), in combination with chemotherapy results in significant improvements in survival and progression-free survival compared with treatment with chemotherapy alone [12–14].

Although combination therapies that include the anti-EGFR antibodies panitumumab and cetuximab have demonstrated clinical efficacy, some patients do not respond to treatment [3–5]. At the time these trials were designed, there were no known biomarkers that predicted response to EGFR-targeted therapies in the treatment of mCRC. Attempts to associate EGFR protein expression with response to cetuximab were unsuccessful [5, 15]. However, activating mutations in *KRAS* (a key component of the EGFR signaling pathway [16]) have subsequently been associated with poor outcomes in patients receiving cetuximab or panitumumab [6, 17–26].

## 2. Preclinical Studies Investigating Combined Vascular Endothelial Growth Factor and Epidermal Growth Factor Receptor Inhibition

Because VEGF and EGFR share downstream signaling components, it has been suggested that there may be potential for additive or even synergistic therapeutic efficacy with therapies targeting both pathways [27]. In mice bearing GEO colon cancer xenografts, simultaneous blockade of VEGF and EGFR with a VEGF antisense oligonucleotide and cetuximab resulted in enhanced antitumor activity and improved survival compared with inhibition of either pathway alone [28]. Similarly, treatment with cetuximab in combination with an anti-VEGF receptor 2 monoclonal antibody resulted in improved antitumor activity in mice with metastases induced by intraperitoneal injection of KM12L4 human colon cancer cells [29]. In a preclinical model of gastric cancer, inhibition of VEGF and EGFR signaling resulted in significantly improved inhibition of tumor growth [30]. Some evidence suggests that this improved inhibition in preclinical studies may have been due to interactions between the VEGF and EGFR signaling pathways. For example, treatment with an anti-EGFR monoclonal antibody was shown to inhibit VEGF production; whereas treatment with vandetanib (an inhibitor of the tyrosine kinase activity of VEGF receptors) blocked epidermal growth factor-induced EGFR phosphorylation [31, 32].

## 3. Phase II Combination Studies of Vascular Endothelial Growth Factor Inhibitors and Epidermal Growth Factor Receptor Inhibitors

Encouraging results have been obtained in phase II studies that investigated regimens incorporating chemotherapy in combination with bevacizumab and an EGFR inhibitor in the treatment of mCRC. BOND-2 was a small (*N* = 83), randomized, phase II trial that evaluated the safety and efficacy of cetuximab and bevacizumab with or without irinotecan in patients with irinotecan-refractory mCRC [33]. The patient population enrolled in the trial had received extensive previous treatment; the median number of prior chemotherapy regimens was 3. Patients in arm A (*n* = 43) of the study received cetuximab, bevacizumab, and irinotecan; whereas patients in arm B (*n* = 40) received only cetuximab plus bevacizumab. Treatment continued until disease progression per Response Evaluation Criteria in Solid Tumors (RECIST) criteria, unacceptable toxicity, or the patient withdrew consent. Patients receiving cetuximab, bevacizumab, and irinotecan had a time to tumor progression of 7.3 months, a response rate of 37%, and an overall survival of 14.5 months. In comparison, patients receiving cetuximab and bevacizumab without irinotecan had a time to tumor progression of 4.9 months, a response rate of 20%, and an overall survival of 11.4 months. These response rates compare favorably with those from other studies in patients with refractory mCRC. In the BOND-1 study, patients with irinotecan-refractory mCRC treated with cetuximab with (*n* = 218) or without (*n* = 111) irinotecan had response rates of 23% and 11%, respectively [5]. In the EPIC study, patients with mCRC refractory to fluoropyrimidine and oxaliplatin treatment received irinotecan with (*n* = 648) or without (*n* = 650) cetuximab and had response rates of 16% and 4%, respectively [4]. In a study of bevacizumab in combination with oxaliplatin, patients with mCRC refractory to fluoropyrimidine and irinotecan were treated with FOLFOX plus bevacizumab (*n* = 286), FOLFOX alone (*n* = 291), or bevacizumab alone (*n* = 243). Overall response rates in these treatment groups were 23%, 9%, and 3%, respectively [12]. Finally, patients treated with irinotecan (CPT-11) as a second-line therapy had overall response rates ranging from 13% to 25% [34, 35]. 

In addition to the encouraging activity observed in BOND-2, the incidence of toxicity attributable to cetuximab, which consisted chiefly of skin rash, was similar with and without irinotecan, suggesting that the addition of irinotecan to the cetuximab/bevacizumab combination did not exacerbate toxicity. However, because of the trial design, no comparison of the toxicity or antitumor activity of this combination with the combination of bevacizumab and irinotecan could be made. 

In another small (*N* = 35) phase II trial, Meyerhardt et al. investigated the efficacy and safety of a combination of FOLFOX chemotherapy, bevacizumab (5 mg/kg intravenously), and erlotinib (150 mg/d orally) in the first-line treatment of patients with mCRC [36]. This combination resulted in an overall response rate of 34% (95% CI, 18%–50%). In addition to this relatively low response rate (response rates of greater than 47% have been reported in patients receiving bevacizumab plus chemotherapy as a first-line treatment for mCRC [37, 38]), 86% of patients experienced grade 3/4 adverse events. Many withdrew owing to toxicity (51%) or withdrew consent owing to toxic effects (26%), limiting conclusions regarding efficacy. It is unclear whether the use of a small-molecule inhibitor of the EGFR (i.e., erlotinib) may have resulted in differences in antitumor activity and tolerability of this combination compared with use of an anti-EGFR monoclonal antibody in combination with bevacizumab.

Collectively, data from preclinical and phase II clinical studies suggested that combination therapies targeting VEGF and EGFR in combination with chemotherapy might result in improved antitumor efficacy in patients with mCRC but retain acceptable toxicity. However, some results suggest that outcomes using this approach may be different in patients receiving this combination as first-line therapy and highly selected second-line therapy or late-stage patient populations.

Results from several other studies have suggested that inhibition of the VEGF and EGFR pathways may have activity in other tumor types. Combining bevacizumab and an EGFR inhibitor appears to show antitumor activity and acceptable toxicity in the second-line treatment of advanced nonsmall cell lung cancer (NSCLC) [27, 39] and recurrent or metastatic squamous cell carcinoma of the head and neck [40]. Furthermore, treatment with chemotherapy, bevacizumab, and an EGFR inhibitor appears to be a promising treatment approach in patients with advanced NSCLC [41].

## 4. Safety and Efficacy of Regimens Incorporating Chemotherapy, Vascular Endothelial Growth Factor Inhibitors, and Epidermal Growth Factor Receptor Inhibitors in Patients with Metastatic Colorectal Cancer

To date, 2 large phase 3 trials investigating the safety and efficacy of combination therapies incorporating chemotherapy, bevacizumab, and an EGFR inhibitor for first-line treatment of mCRC have recently been completed [42]. These trials were designed based on results from the hypothesis-generating BOND-2 trial, and both were designed and accrued before the identification of *KRAS *mutations as a predictor of poor response to anti-EGFR monoclonal antibodies. In both studies, the combination of chemotherapy, bevacizumab, and an EGFR inhibitor was associated with decreased efficacy. Furthermore, both regimens were associated with substantial toxicity. 

**Figure 1:**
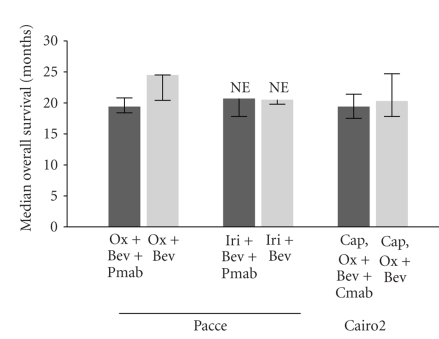
Median overall survival in PACCE and CAIRO2. Bev: bevacizumab; Cap: capecitabine; Cmab: cetuximab; Iri: irinotecan; Ox: oxaliplatin; Pmab: panitumumab.

Panitumumab Advanced Colorectal Cancer Evaluation (PACCE) was a randomized, open-label, phase 3 trial to evaluate patients undergoing first-line treatment for mCRC with either oxaliplatin- or irinotecan-based chemotherapy and bevacizumab, with or without panitumumab [43]. At the time the study was designed, both FOLFOX and FOLFIRI had been shown to be superior to infusional 5-fluorouracil/leucovorin [44, 45], and the 2 regimens appeared to be interchangeable in mCRC [46]. Furthermore, results of the ECOG3200 study had demonstrated improved survival with the combination of bevacizumab plus FOLFOX compared with FOLFOX alone as a second-line therapy in mCRC [12]. Based on these data, oxaliplatin- and irinotecan-based chemotherapy regimens were selected for use in PACCE, with the preponderance of patients (~80%) enrolled in the oxaliplatin-based chemotherapy arm because more substantial data were available supporting the combination of bevacizumab plus oxaliplatin-based chemotherapy and because it was intended that the trial should match the standard of care for first-line treatment of mCRC in community practice. Patients received no panitumumab (*n* = 410) or 6 mg/kg panitumumab (*n* = 413) every 2 weeks in the oxaliplatin/bevacizumab arm and no panitumumab (*n* = 115) or 6 mg/kg panitumumab (*n* = 115) every 2 weeks in the irinotecan/bevacizumab arm. Treatment continued until disease progression or drug intolerability. There was a significant difference in progression-free survival (10.0 versus 11.4 months; hazard ratio [HR], 1.27; 95% CI, 1.06–1.52) and overall survival (19.4 versus 24.5 months; HR, 1.43; 95% CI, 1.11–1.83) in favor of the control group in the oxaliplatin arm (Table 1; Figure 1). In the irinotecan arm, progression-free survival was 10.1 months for the panitumumab group and 11.7 months for the control group (HR, 1.19; 95% CI, 0.79–1.79). Median overall survival in the irinotecan arm was 20.7 months for the panitumumab group and 20.5 months for the control group (HR, 1.42; 95% CI, 0.79–2.62). The response rates were similar between the panitumumab and control groups in both the oxaliplatin arm (46% and 48%, resp.; odds ratio [OR], 0.92; 95% CI, 0.70–1.22) and the irinotecan arm (43% and 40%; OR, 1.11; 95% CI, 0.65–1.90). Grade 3/4 adverse events were increased in both chemotherapy cohorts among patients who received panitumumab. Adverse events occurring more frequently in patients receiving panitumumab compared with the control arm included skin toxicity, diarrhea, dehydration, hypomagnesemia, infections, and pulmonary embolism (Table 2). Treatment with panitumumab in PACCE was terminated early as a result of an observed lack of additional clinical benefit and increased toxicity at a preplanned interim analysis.

CAIRO2 was a randomized, open-label, phase 3 trial that evaluated the efficacy and safety of bevacizumab and capecitabine/oxaliplatin with or without cetuximab as a first-line treatment in 755 patients with mCRC [47]. Patients received capecitabine and oxaliplatin in combination with either bevacizumab (*n* = 368) or bevacizumab and cetuximab (*n* = 368). To prevent potential neurotoxicity, patients received a maximum of 6 cycles of oxaliplatin. Although it is unclear how many patients received oxaliplatin at a later time, 48 patients received oxaliplatin following disease progression, including 18 in the control group and 30 in the cetuximab group. Patients receiving chemotherapy, bevacizumab, and cetuximab had a decreased progression-free survival time, the primary endpoint of the study, compared with patients receiving chemotherapy and bevacizumab (9.4 versus 10.7 months, resp.; HR, 1.22; 95% CI, 1.04–1.43; see Table 1). The response rate and median overall survival were not significantly different between the cetuximab and control groups (52.7% versus 50.0% and 19.4 versus 20.3 months, resp.; see Figure 1). The overall incidence of grade 3/4 toxicity was significantly increased in patients treated with chemotherapy, bevacizumab, and cetuximab compared with patients receiving chemotherapy and bevacizumab. The most frequent grade 3/4 toxicities were diarrhea, acneiform rash, hand-foot skin reaction, fatigue, hypertension, and sensory neuropathy (Table 2).

## 5. Biomarkers for the Prediction of Response in Regimens Incorporating Chemotherapy, Vascular Endothelial Growth Factor Inhibitors, and Epidermal Growth Factor Receptor Inhibitors

There has been growing interest in the development of biomarkers for the identification of patients most likely to respond to therapy. At the time PACCE and CAIRO2 were initiated, mutations of codons 12 and 13 of the *KRAS* gene had been associated with poor prognosis in mCRC [48–50]. Furthermore, *KRAS* mutations were subsequently found to be associated with poor response to either panitumumab or cetuximab either alone or in combination with chemotherapy [6, 17–26]. For example, in a study of panitumumab plus best supportive care compared with best supportive care alone in second-line treatment of mCRC, clinical benefit was confined to patients with wild-type *KRAS *[17]. The response rate among patients with wild-type *KRAS* was 17%, whereas no patient with mutant *KRAS* had a response.

In PACCE, *KRAS* mutations (codons 12 or 13) were identified in 346 of 865 (40%) samples evaluated (39% in the oxaliplatin cohort and 43% in the irinotecan cohort) [43]. Progression-free survival was longer among patients who did not receive panitumumab in both chemotherapy cohorts among patients with both wild-type and mutant *KRAS* (Table 3). In the oxaliplatin-based chemotherapy cohort, response rates were similar between arms in both *KRAS* subsets (Table 3). Surprisingly, overall survival favored the control group in the wild-type subset of the oxaliplatin-based chemotherapy cohort (24.5 versus 20.7 months; HR, 1.89; 95% CI, 1.30–2.75). In the irinotecan-based chemotherapy cohort, response rates were higher in the wild-type *KRAS* subset among patients who received panitumumab compared with those in the control group (54% versus 48%). In the mutant *KRAS* subset, response rates were lower among patients who received panitumumab (30% versus 38%). Overall survival favored the control arm and was not influenced by *KRAS* status in the irinotecan-based chemotherapy cohort.

In CAIRO2, *KRAS* mutations were identified in 206 of 528 (39.6%) patients [47]. Forty-one percent of patients in the control arm and 38% of patients in the cetuximab arm had mutations in *KRAS*. Progression-free survival was similar between the cetuximab and control arms in the wild-type *KRAS* subset but favored the control arm in the mutant *KRAS* subset (12.5 versus 8.1 months). There was no association between *KRAS* status and overall survival in either arm (Table 3). Thus, unlike previous studies, which have demonstrated that *KRAS* mutations predict a poor response to EGFR inhibitors, *KRAS* mutational status in PACCE and CAIRO2 failed to predict patient response to an anti-EGFR antibody when administered in combination with bevacizumab and chemotherapy.

Associations between response in BOND-2 and germline polymorphisms in a variety of genes have been investigated. It was found that polymorphisms in *EGF*, the gene that encodes EGF*,* were associated with tumor response in patients who received cetuximab, bevacizumab, and irinotecan [51]. *EGF* polymorphisms affect EGF expression [52] and have been associated with improved survival in mCRC patients treated with cetuximab and irinotecan [53].

## 6. Discussion

Collectively, the results of these trials suggest that the use of combination therapies consisting of bevacizumab, an EGFR-specific antibody, and chemotherapy in the first-line treatment of mCRC is associated with negative risk benefit. This combination was not associated with improved outcomes in patients receiving panitumumab and in patients receiving cetuximab, suggesting a class effect of EGFR inhibitors rather than a specific effect of either of the agents. Although there may be differences in the safety profiles of panitumumab and cetuximab, the similarity of the results of PACCE and CAIRO2 suggests that, in the context of combination therapy with bevacizumab and chemotherapy, their similarities outweigh their differences. These results were surprising given the results of the BOND-2 study in which the combination of cetuximab and bevacizumab with irinotecan appeared to be effective in patients with irinotecan-refractory mCRC. The difference in the results of BOND-2 and PACCE/CAIRO2 studies may reflect differences in patient selection among the 3 trials.

Overall, the results of PACCE and CAIRO2 were similar. In both studies, the combination of an EGFR-specific monoclonal antibody, bevacizumab, and chemotherapy resulted in significantly reduced progression-free survival and exacerbation of toxicity compared with treatment with bevacizumab and chemotherapy. In the oxaliplatin arm of PACCE (but not the irinotecan arm), a significant decrease in overall survival was observed. This decrease in survival might have been due to reduced chemotherapy dose intensity in the panitumumab arms. Although no decrease in overall survival was observed in patients who received cetuximab in CAIRO2, this was not the primary endpoint of the study, and it is unclear whether the study was powered to detect a change in overall survival in the 2 arms. It is also interesting to note that overall survival in the bevacizumab/oxaliplatin arm (24.5 months) was relatively long compared with survival in other phase III studies that have investigated bevacizumab in combination with oxaliplatin-based chemotherapy, including the NO16966 trial (21.3 months) [38] and CAIRO2 (20.3 months) [47]. The results of PACCE and CAIRO2 are interesting given that treatment with panitumumab, cetuximab, or bevacizumab in combination with chemotherapy has antitumor activity and acceptable toxicity in mCRC [3–6, 12–14]. There are several potential explanations for the exacerbated toxicities. First, there may have been a pharmacodynamic interaction between the agents. For example, treatment with an EGFR-specific antibody may have altered downstream signaling required for bevacizumab or chemotherapy activity, or alternatively, bevacizumab may have altered signaling activity required for anti-EGFR monoclonal antibody activity. Second, there may have been trial design factors that influenced toxicity. Potentially, toxicity reporting bias due to the open-label design may have contributed to the difference in adverse events in the treatment arms. Alternatively, treatment discontinuation in patients without disease progression may have reduced the ability to observe some treatment effects. Finally, there may have been pharmacokinetic interactions between the agents. 

Although these results suggest that VEGF/EGFR/chemotherapy combination regimens are problematic in mCRC, the lack of benefit associated with each regimen in first-line therapy should not be generalized to each of the individual agents or to any of the chemotherapy-plus-monoclonal antibody combinations. In particular, the exacerbation of toxicity observed should not be ascribed to either cetuximab or panitumumab merely because these were the agents added to the bevacizumab plus chemotherapy combination, and the trial designs did not include panitumumab plus chemotherapy or cetuximab plus chemotherapy arms. To date, panitumumab combined with chemotherapy regimens has been well tolerated. Recent results from 2 large phase 3 trials for first- and second-line mCRC have shown that panitumumab has acceptable toxicity in combination with chemotherapy. In phase 2 studies, treatment with panitumumab with chemotherapy has shown encouraging antitumor activity [3, 54]. Cetuximab is approved for use in combination with irinotecan for second-line treatment of mCRC [55]. 

Because of the encouraging results in BOND-2, PACCE and CAIRO2 were conducted before additional data were gathered from phase 1, 2, or randomized phase 2 studies. Notably, BOND-2 was composed of a treatment arm consisting of only biologics without chemotherapy and a treatment arm consisting of biologics and irinotecan only. At present, the contribution of different chemotherapy regimens in PACCE (leucovorin/5-fluorouracil/irinotecan, or leucovorin/5-fluorouracil/oxaliplatin) or CAIRO2 (capecitabine/oxaliplatin) contributed to the observed toxicity. Moreover, it is unknown whether alternative chemotherapy combinations may have resulted in better outcomes. 

Reasons for variability in observed associations between *KRAS* status and response and overall survival in PACCE and CAIRO2 remain unclear [43, 47]. Even among patients with wild-type *KRAS*, the negative interaction between bevacizumab and panitumumab appeared to counteract this potential advantage. However, it should be noted that, in PACCE, *KRAS* data were from an unplanned retrospective subset analysis and are not definitive. Some comparisons may represent chance findings resulting from the small sample size. Differential exposure to EGFR inhibitors in later lines of therapy for the control arm of PACCE might have affected survival outcomes in the *KRAS* wild-type group. Biomarkers in addition to *KRAS* may have also influenced outcome. *BRAF* mutations (V600E) [18, 56], *PTEN* gene amplification [57], and EGFR ligand expression levels [19] have been identified as potential predictive markers for EGFR-targeted therapies in mCRC [18, 19]. A recently published retrospective analysis of tumor samples from CAIRO2 demonstrated that *BRAF* mutations were associated with poor clinical outcome [58]. Of the 516 tumors available for analysis, 45 (8.7%) tumors had *BRAF* V600E mutations. Consistent with previous studies [56, 59], *BRAF* mutations were observed only in tumors with wild-type *KRAS*. Across both treatment arms in the study, the presence of *BRAF* mutations was associated with significant reductions in median overall survival and progression-free survival compared with wild-type *BRAF* but the response rate was unaffected [58]. However, the presence or *BRAF* mutations did not appear to be associated with poor response to cetuximab, suggesting that *BRAF *may be a better predictor of prognosis than of response to VEGF/EGFR inhibition. The contribution of potential biomarkers such as *BRAF* mutations to the outcome of patients is unknown. Furthermore, it is unknown whether there were imbalances in these markers across the treatment arms in PACCE. Results of pharmacogenomic analyses of BOND-2 suggest that biomarkers that predict response in this combination might be found [51]; however, at present this hypothesis remains untested. 

The results of PACCE and CAIRO2 are likely to affect the conduct and design of current and future studies. In particular, the open-label, phase III CALGB 80405 study was designed to determine if cetuximab with or without bevacizumab in combination with chemotherapy (FOLFOX or FOLFIRI) improves overall survival in the first-line treatment of patients with mCRC [60]. Enrollment in arm C (chemotherapy with bevacizumab and cetuximab) was suspended by the sponsor. Concerns raised by PACCE and CAIRO2 have also resulted in the suspension of the randomized phase III SWOG 0600 study, which was designed to investigate irinotecan-based chemotherapy and cetuximab with or without bevacizumab for the treatment of patients with mCRC refractory to bevacizumab in combination with chemotherapy. Interestingly, preliminary results of the BOND 2.5 study, which assessed cetuximab and bevacizumab in combination with irinotecan-based chemotherapy in patients with mCRC refractory to bevacizumab in combination with irinotecan-based chemotherapy, suggested that although the combination had acceptable toxicity, response rates among these patients appeared inferior to that observed in BOND-2 [61]. The objective response rate was 9% and median time to progression was 3.9 months. These results suggest that patient selection may be of critical importance in the design of trials investigating combinations of chemotherapy and EGFR inhibitors with bevacizumab.

## 7. Conclusions

The results of PACCE, CAIRO2, and other trials do not support the use of therapies consisting of chemotherapy in combination with a VEGF inhibitor and an EGFR inhibitor as a first-line therapy for mCRC outside investigational studies. Although it is possible that additional investigational studies may identify combinations that provide clinical benefit in either the first- or second-line settings, it will be necessary to carefully design and monitor these studies given the exacerbated toxicity observed in PACCE and CAIRO2. Careful patient selection is likely to be a key determinant of any future success of VEGF/EGFR regimens. Despite the results of PACCE and CAIRO2, it must be noted that combination therapy with chemotherapy and bevacizumab or chemotherapy and panitumumab or cetuximab is still associated with therapeutic benefit in mCRC.

## Figures and Tables

**Table 1:** Comparison of efficacy results from the PACCE and CAIRO2 studies.

	PACCE oxaliplatin cohort [43]		CAIRO2 [47]	
	(*N* = 823)		(*N* = 755)	
	Ox + Bev + Pmab	Ox + Bev	Cap, Ox + Bev + Cmab	Cap, Ox + Bev
Median progression-free survival (95% CI), mo	10.0 (8.9–11.0)	11.4 (10.5–11.9)	9.4 (8.4–10.5)	10.7 (9.7–12.3)
Progression-free survival events, *n* (%)	246 (60)	221 (54)	316 (84)	293 (78)
Overall response rate, %*	46	48	50.0	52.7
Median overall survival (95% CI), mo	19.4 (18.4–20.8)	24.5 (20.4–24.5)	20.3 (17.8–24.7)	19.4 (17.5–21.4)
Deaths, *n* (%)	143 (35)	108 (26)	214 (57)	193 (51)

Bev: bevacizumab; Cap: capecitabine; Cmab: cetuximab; NR: not reported; Ox: oxaliplatin; PACCE: Panitumumab Advanced Colorectal Cancer Evaluation; Pmab: panitumumab.

*Overall response rate = complete response + partial response.

**Table 2:** Comparison of adverse events of interest in the PACCE and CAIRO2 studies.

	PACCE oxaliplatin cohort [43]				CAIRO2 [47]	
	(*N* = 823)				(*N* = 755)	
	Ox + Bev + Pmab		Ox + Bev		Cap, Ox + Bev + Cmab	Cap, Ox + Bev
	Grade 3	Grade 4	Grade 3	Grade 4	Grade 3/4	Grade 3/4
Incidence of toxicity, %						

Skin toxicity	35	1	1	0	39.1	20.8
Diarrhea	22	2	12	1	26.0	19.1
Infection	16	2	8	2	6.0	6.8
Hypertension	4	0	5	0	9.3	14.8
Hypomagnesemia	3	1	0	0	NR	NR
Neuropathy*	3	<1	7	0	7.7	10.4
Nausea/vomiting^†^	13	0	6	1	6.3/6.0*	8.5/8.2*
Deep vein thrombosis	7	0	8	0	NR	NR
Pulmonary embolism	0	6	0	4	NR	NR
Venous thromboembolic events	NR	NR	NR	NR	8.2	6.8
Arterial thromboembolic events	NR	NR	NR	NR	2.2	3.3

Bev: bevacizumab; Cap: capecitabine; Cmab: cetuximab; NR: not reported; Ox: oxaliplatin; PACCE: Panitumumab Advanced Colorectal Cancer Evaluation; Pmab: panitumumab.

*Neuropathy events were reported as “sensory neuropathy” in CAIRO2.

^†^The incidence of nausea and vomiting was reported together in PACCE but as separate adverse events in CAIRO2. The first number indicates the reported incidence of nausea and the second number indicates the incidence of vomiting.

**Table 3:** Association between *KRAS* status and efficacy in PACCE and CAIRO2.

	Response rate, %		Progression-free survival, mo		Overall survival, mo	
	Wild-type	Mutant	Wild-type	Mutant	Wild-type	Mutant
	*KRAS*	*KRAS*	*KRAS*	*KRAS*	*KRAS*	*KRAS*
PACCE oxaliplatin cohort [43] (*N* = 664)						
Ox + Bev + Pmab	50	47	9.8	10.4	20.7	19.3
Ox + Bev	56	44	11.5	11.0	24.5	19.3
CAIRO2 [47] (*N* = 528)						
Cap, Ox + Bev + Cmab	61.4	45.9	10.5	8.1	21.8	17.2
Cap, Ox + Bev	50.0	59.2	10.6	12.5	22.4	24.9

Bev: bevacizumab; Cap: capecitabine; Cmab: cetuximab; NR: not reported; Ox: oxaliplatin; PACCE: Panitumumab Advanced Colorectal Cancer Evaluation; Pmab: panitumumab.
